# The Genetics of Sleep Disorders in Children: A Narrative Review

**DOI:** 10.3390/brainsci11101259

**Published:** 2021-09-23

**Authors:** Greta Mainieri, Angelica Montini, Antonio Nicotera, Gabriella Di Rosa, Federica Provini, Giuseppe Loddo

**Affiliations:** 1 Department of Biomedical and Neuromotor Sciences, University of Bologna, 40138 Bologna, Italy; greta.mainieri2@unibo.it (G.M.); angelica.montini@studio.unibo.it (A.M.); 2 Unit of Child Neurology and Psychiatry, Department of Human Pathology of the Adult and Developmental Age, “Gaetano Barresi” University of Messina, 98124 Messina, Italy; antonionicotera@ymail.com (A.N.); gabriella.dirosa@unime.it (G.D.R.); 3 IRCCS Istituto Delle Scienze Neurologiche di Bologna, 40139 Bologna, Italy; 4 Azienda USL di Bologna, 40124 Bologna, Italy; loddogiuseppe0@gmail.com

**Keywords:** insomnia, sleep-related breathing disorders, hypersomnolence, circadian rhythm sleep-wake disorders, parasomnias, sleep-related movement disorders

## Abstract

Sleep is a universal, highly preserved process, essential for human and animal life, whose complete functions are yet to be unravelled. Familial recurrence is acknowledged for some sleep disorders, but definite data are lacking for many of them. Genetic studies on sleep disorders have progressed from twin and family studies to candidate gene approaches to culminate in genome-wide association studies (GWAS). Several works disclosed that sleep-wake characteristics, in addition to electroencephalographic (EEG) sleep patterns, have a certain degree of heritability. Notwithstanding, it is rare for sleep disorders to be attributed to single gene defects because of the complexity of the brain network/pathways involved. Besides, the advancing insights in epigenetic gene-environment interactions add further complexity to understanding the genetic control of sleep and its disorders. This narrative review explores the current genetic knowledge in sleep disorders in children, following the International Classification of Sleep Disorders—Third Edition (ICSD-3) categorisation.

## 1. Introduction

Sleep is a universal, essential behaviour, persistent throughout evolution and across different species [1]. In humans, the regular development of a mature sleep system from childhood to adulthood is an important milestone, with potential consequences for both neurological and health concerns [2]. Consequently, the prompt identification of sleep disorders in children is of the utmost importance, in order to address potential sequelae, treat comorbid conditions and relieve the burden on other family members [2]. Sleep is a dynamic process and could be influenced by external factors (environment, medical comorbidities, drugs), whose effect may possibly lead to some sleep disorders [3,4]. On the other hand, several sleep disorders recognise a genetic predisposition and the current expansion of advanced molecular techniques might serve for a better identification and consequent treatment of the different disorders [5,6,7,8,9]. Over the last decades, a bidirectional relationship between genetic factors and sleep has been recognised; specific genes may influence circadian regulation, neurotransmission and signalling pathways involved in sleep processes, whereas sleep may affect, in turn, gene expression [10,11,12]. Moreover, a differentiated gene expression during sleep and wakefulness is recognised, mostly depending on the different biological functions of the two states. In fact, genes encoding proteins involved in energy metabolism and oxidative cellular response, upregulated during wakefulness, leave the place to genes regulating protein biosynthesis, synaptic downscaling and cell membrane processes during sleep [13]. Sleep has proved to play a fundamental role in synaptic plasticity, with implications in the achievement and upholding of recently learned motor and cognitive skills, especially relevant for paediatric populations [2,14]. Moreover, the inter-relationships between sleep disorders and other neurological and psychiatric conditions, on the one hand, and metabolic/cardiovascular disturbances, on the other, are becoming increasingly clear, with implications both in children and adults [6,15,16,17,18,19,20]. Genetic studies are showing that a common genetic background might play a pivotal role, constituting a linking point among these different conditions.

In this narrative review, we specifically focused on the principal sleep disorders affecting paediatric populations, following the six main categories of the International Classification of Sleep Disorders—Third Edition (ICSD-3): Insomnia, Sleep-related Breathing Disorders, Central Disorders of Hypersomnolence, Circadian Rhythm Sleep-Wake Disorders, Parasomnias and Sleep-Related Movement Disorders [21]. Among the different sleep disorders, genetic components assume weights of variable importance. The breakthrough of genome-wide association studies (GWAS) has consistently expanded the genetic literature, also as regards sleep disorders. The aim of our paper is to summarise the current evidence of the genetics of sleep disorders, including the most recent GWA studies. To our knowledge, this is the first paper attempting to provide the present state of the art of the genetic aspects of all sleep disorders in paediatric populations. We emphasise the possible connections between genetics and the different sleep conditions, together with the relationships with neuropsychiatric or metabolic disorders.

## 2. Methods

A narrative literature research has been performed on Pubmed and Scopus research databases, with no restriction on articles’ publication date. Published papers up to July 2021 were included. We also included case series or case reports, when relevant genetic findings were reported. We used the following keywords: “insomnia” AND (“genetics” or “genes”), “sleep-related breathing disorders” AND (“genetics” or “genes”), “central disorders of hypersomnolence” AND (“genetics” or “genes”), “circadian rhythm sleep-wake disorders” AND (“genetics” or “genes”), “parasomnias” AND (“genetics” or “genes”), “sleep-related movement disorders” AND (“genetics” or “genes”). We only considered works in English language. Additional papers were inserted using the references of the selected works, when relevant.

## 3. Insomnia

Insomnia is defined as the difficulty in initiating and/or maintaining sleep, early awakening or poor sleep quality in the presence of adequate opportunity and circumstance for sleep. Daytime consequences include reduced scholar performance and social dysfunction due to mental (impaired attention, concentration or memory), mood (decreased mood or irritability) and behavioural disturbance (hyperactivity, impulsivity or aggression) [21]. Chronic insomnia affects 20% to 30% of children [21,22], reaching a prevalence of up to 86% in those with neurodevelopmental disabilities [23]. Among the different subtypes included in chronic insomnia (psychophysiological, idiopathic, paradoxical insomnia, inadequate sleep hygiene, behavioural insomnia of childhood), two, in particular, are typical of infancy. “*Idiopathic insomnia*” occurs by definition in infancy or early childhood [21]. Even if no consistent genetic markers have been identified, idiopathic insomnia is presumed to have a genetically determined base or congenital alterations in the sleep-inducing or arousal system [21]. The “*behavioural insomnia of childhood*” is divided into “sleep onset association” and “limit setting” subtypes. The first one derives from altered learned behaviours (such as the need to be rocked or to eat) or the child’s dependency on a specific environment (car, light, etc.) or objects, whereas the “limit-setting” type is characterised by bedtime restriction due to the lack of appropriate parental rules [21,24]. The diagnosis of insomnia disorder is clinical. Video-polysomnography (VPSG) may be warranted if a sleep breathing disorder or parasomnia/sleep-related complex motor behaviours are suspected [21].

The relationship between insomnia and genetic factors is intricate. The insomniac phenotype is thought of as a consequence of inheriting many alleles that exert small but cumulative effects on sleep regulatory circuits [25], associated with the influence of epigenetic mechanisms [26]. Data from twin and family studies indicate a degree of heritability ranging from 14% to 71% in children [27,28,29]. Several gene polymorphisms involved in the circadian clock, immune regulation and neurotransmitter pathways have been identified. Polymorphisms in circadian genes (*CLOCK gene*, *PER3*, *peroxisome proliferator-activated receptor-c coactivator-1a*) [30,31,32,33] and neuromodulators involved in the sleep-wake regulation have been associated with insomnia, sometimes in association with depressive symptoms, especially when serotoninergic pathways were involved [34,35,36,37]. From GWAS, several other polymorphisms have been linked to insomnia or prolonged sleep latencies, such as those in genes involved in bipolar disorder and schizophrenia (*ROR1* and *PLCB1*) [38], a subunit of voltage-dependent calcium channels [39], circadian genes (*ULF1*) [40] and GABA and monoamine signalling [41]. In addition, a strong association with the *MEIS 1* locus, strongly related with restless leg syndrome (RLS) [42] was identified, suggesting a pleiotropic effect of this gene and implications in phenotypic overlap (RLS comorbid with insomnia) [15,16]. To date, the largest GWAS included 1,331,010 participants and identified 202 genomic risk loci for insomnia, pointing out a causal effect of insomnia on depression, diabetes and cardiovascular disease. Interestingly, this study evaluated the different gene expression in different types of brain cells and revealed an enrichment of risk genes for insomnia in cortical areas as well as the striatum, supporting the involvement of the striato-cortical network in insomnia pathophysiology [17].

In summary, risk genes for insomnia have been associated with circadian clock genes, pathways regulating sleep-wake mechanisms and psychiatric and cardio-metabolic conditions. The identification of insomnia risk genes would be helpful to clarify the neurobiological mechanisms of insomnia. Furthermore, it could widen the opportunity for insomnia prevention or early and specific treatment, screening genes that influence the individual response to certain drugs.

## 4. Sleep-Related Breathing Disorders

Sleep-related breathing disorders are characterised by irregularities of respiration during sleep and are grouped into obstructive sleep apnoea and central sleep apnoea disorders, sleep-related hypoventilation disorders and sleep-related hypoxemia disorders [21].

*Obstructive Sleep Apnea Syndrome* (OSAS) is characterised by repetitive episodes of complete (apnea) or partial (hypopnea) upper airway obstruction occurring during sleep. These events often result in a reduction in blood oxygen saturation and are usually concluded by brief arousals from sleep [21]. OSAS prevalence in children ranges from 1 to 5.7% [21,43] and could be associated with excessive daytime sleepiness (especially in older children), developmental, behavioural and learning issues including attention disturbances, hyperactivity, moodiness, irritability and impaired performance [21]. Diagnostic criteria include the evidence of one or more obstructive/mixed apnoeas, or hypopnea, per hour of sleep on polysomnography or a pattern of obstructive hypoventilation, defined as at least 25% of total sleep time with hypercapnia (PaCO2 > 50 mm Hg) in association with one or more of the following: snoring; paradoxical or obstructed breathing during sleep; sleepiness, hyperactivity, behavioural or learning problems [21]. Polysomnography is the standard diagnostic test for the diagnosis of OSA in children [44].

**Figure 1:**
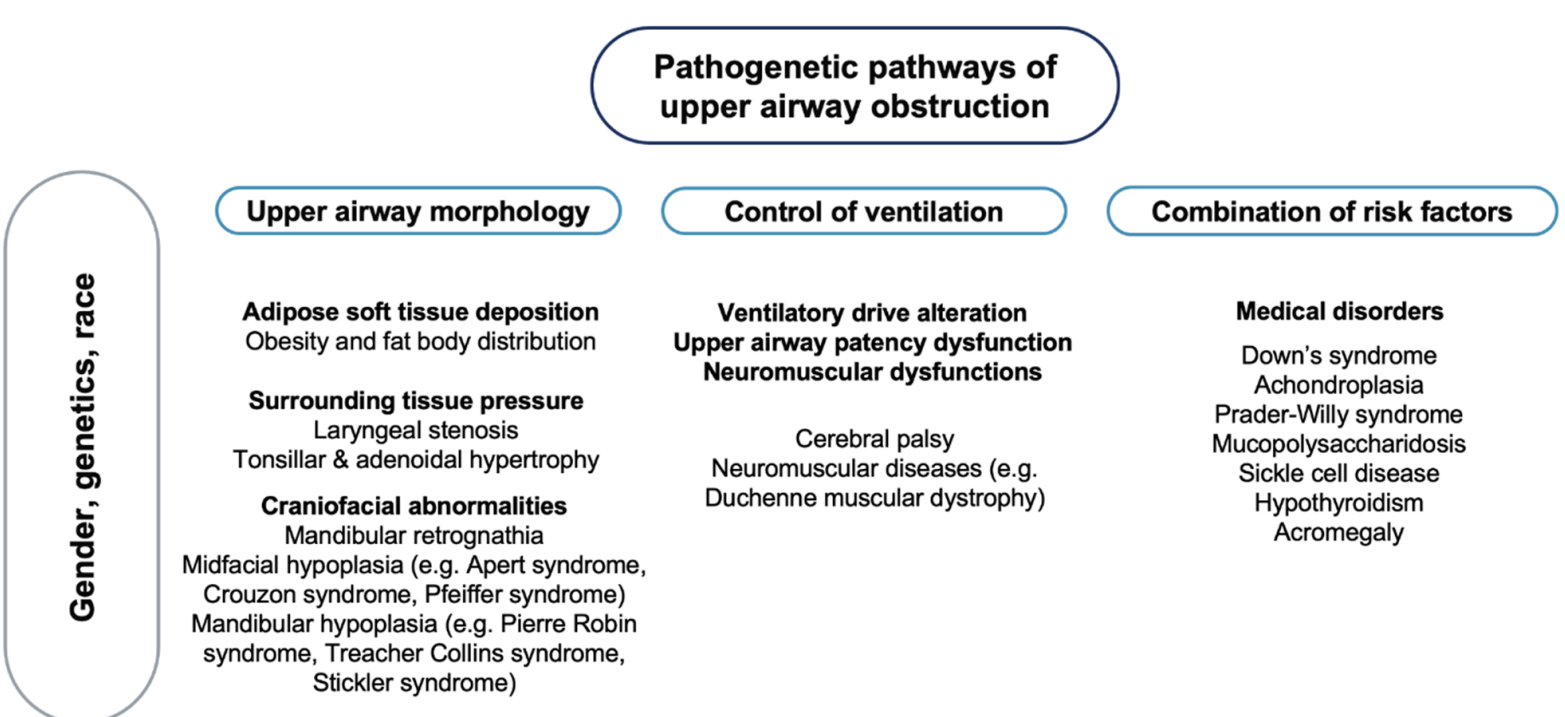
Pathogenetic pathways of upper airway obstruction. **Note**: On a background influenced by gender, genetics and race, different pathogenetic factors may lead to upper airway obstruction.

OSA is characterised by a complex phenotype, involving different pathogenetic factors [45] (Figure 1), with no recognised monogenic predisposition [46].

Most cases of OSA do not exhibit classical Mendelian patterns of inheritance, suggesting multifactorial pathogenesis, where many common variants with small or moderate genetic effects determine disease heritability [47] as well as different phenotypic expressions of the disorder. In a study on 445 first-degree relatives of 115 children with OSA, 26.6% of the adult and 12.2% of the paediatric relatives had symptoms suggestive of OSA, supporting the hypothesis of a possible role of a genetic mechanism in the aetiology of this syndrome [48]. It has been speculated that the craniofacial structure, body habitus and ventilatory control mechanism play a role in the pathogenesis of OSA [45]. The involvement of genetic factors in the craniofacial phenotypic dysmorphology associated with breathing dysfunctions has been reported in a study on 50 children with Class III malocclusion, disclosing silent mutations in the *PHOX2B* genotype in 32% of patients and none of the controls [49]. The *PHOX2B* gene is involved in human development, especially in the neural crest, a group of cells in the early embryo from which many tissues in the face and skull originate [50]. Furthermore, several neural crest cells migrate to form parts of the autonomic nervous system, which controls many functions and, above all, breathing [51]. Genetic factors involved in metabolic pathways and cognitive functions also seem to play a relevant role in the pathogenesis of OSA in children. In a study on 229 children with and without apnoea and 412 relatives, being overweight had a significant modification effect on the familial aggregation and the heritability [52]. The link between OSA in children and being overweight can be explained by different mechanisms. Children with OSA have been reported to have an increased expression of the *Liver X receptors* (*LXRs*), nuclear receptors that play a central role in the transcriptional control of lipid metabolism [53], as well as higher plasma fatty-acid binding protein 4, a cytosolic protein abundantly expressed in adipocytes and macrophages [54]. Contrasting data have been reported on the association between OSA and *Apolipoprotein E* (*APOE*) gene [47]. The *APOE* gene is involved in lipid metabolism, but it also has three common alleles with effects on the nervous system [55]. Of these, the *APOE ε4* allele has been more frequently reported in children with OSA and particularly in those who develop neurocognitive deficits [56]. In addition, polymorphisms within the *NADPH oxidase* (*NOX*) gene or its functional subunits may account for important components of the variance in cognitive deficits associated with OSA in children [57]. *NOX* has been shown to mediate neural cell loss in the context of intermittent hypoxia during sleep [58]. A genome-wide gene expression study in non-obese children with OSA showed an altered gene expression in circulating leukocytes regulating and modulating the inflammatory response [59]. Other studies have underlined the association between paediatric OSA and genes involved in endothelial dysfunction and inflammatory responses (*Forkhead box P3*, *interferon regulatory factor 1*, *macrophage migration inhibitory factor*, *endothelial nitric oxide synthase* and *interleukin-10*) [60,61,62,63,64].

In conclusion, both in children and adults, genetic factors may influence anatomical structures or metabolic pathways associated with OSAS. However, the pathogenesis of OSAS is multifactorial, and further studies are needed to better characterise the genetic role. *Central sleep apnoea* (CSA) is defined as the absence of chest and abdominal movements associated with a cessation of airflow for more than 20 s, or lasting more than two baseline respiratory cycles if associated with an arousal, awakening or oxygen desaturation of at least 3% [65]. The ICSD-3 distinguishes CSA with Cheyne–Stokes breathing (which will not be discussed in this review because it occurs in individuals over 60), CSA due to a medical disorder without Cheyne–Stokes respiration, CSA due to high-altitude periodic breathing, CSA due to a medication or substance, treatment-emergent CSA, primary or idiopathic CSA (usually occurring in middle-aged/elderly individuals), primary sleep apnoea of infancy and prematurity [21].

In newborns and infants, CSAs are physiological, especially in the context of a sigh, movement and/or REM sleep [66]. In otherwise healthy 1–18-year-old individuals, the prevalence of central apnoeas, lasting 10–18 s, reached 30% [67]. Primary CSA of infancy is characterised by apnoea or cyanosis in infants of at least 37 weeks with recurrent, prolonged central apnoeas lasting ≥20 s with periodic breathing for ≥5% of total sleep time on polysomnographic recordings [21]. Similarly, primary CSA of prematurity affects infants of less than 37 weeks. Both disorders can be associated with hypoxemia and bradycardia [21], with a risk of neurodevelopmental disturbances [68]. A genetic basis on twins with primary CSA of prematurity has been described [69], but conclusive data are lacking for most CSA subtypes. Genes involved in hypoxic response such as the Endothelial PAS domain-containing protein 1 (EPAS1) or the Hypoxia inducible factors (HIF1A and HIF2A) have been linked to CSA due to high-altitude periodic breathing, because of a possible role in the genetic adaptation of high-altitude hypoxia [70,71]. In the paediatric population, CSA occurs more commonly in association with an underlying medical disorder, especially the Arnold–Chiari malformation, brainstem disease or syndromic conditions [72]. Indeed, a genetic contribution in CSA is mostly related to these secondary forms. Specifically, genetic factors linked to secondary CSA are those involved in neuromuscular disorders [66], Trisomy 21, Joubert syndrome [72], Prader–Willi syndrome [73], achondroplasia [74] and other rare genetic diseases, namely spondyloepiphyseal dysplasia congenita, Pierre Robin syndrome with Cornelia de Lange syndrome and Potocki–Lupski syndrome [73,75].

*Sleep-related hypoventilation disorders* are characterised by insufficient sleep-related ventilation resulting in an abnormally elevated arterial partial pressure of CO2 during sleep [21]. The *congenital central alveolar hypoventilation syndrome* (CCHS) is a rare genetic condition characterised by alveolar hypoventilation due to a deficient autonomic central control of ventilation and a diffuse autonomic dysfunction [76]. Cyanosis, feeding difficulties, hypotonia or central apnoea are common features of the disorder. Different mutations of the *PHOX2B* gene (mostly heterozygous) have been reported in CCHS. This gene encodes a transcription factor expressed in the developing hindbrain and peripheral nervous system as well as in all noradrenergic and visceral motor and branchiomotor neurons of the cranial nerves. Its expression has also been detected in neuronal groups involved in the medullary control reflexes of autonomic functions [77]. The most frequent mutation is an in-frame tandem duplication of tracts of different lengths of the polyalanine stretch in the exon 3 [51,77,78,79]. The number of duplications is negatively correlated to anthropometric measures such as mandible breadth, nasolabial angle, lateral lip height and mandible-face width index [80]. Sleep-related hypoventilation and autonomic dysregulation in the absence of *PHOX2B* gene mutation is encountered in *Late-Onset Central Hypoventilation with hypothalamic dysfunction* (ROHHAD) [21]. This disorder affects patients aged 2–3 years old and is characterised by at least two of the following: obesity, endocrine abnormalities of hypothalamic origin, severe emotional or behavioural disturbances, tumours of neural origin [21]. The mortality rate is estimated at 50–60% due to hypoventilation, cardiopulmonary failure and cardiopulmonary arrest [81]. The similarities between ROHHAD and CCHS suggest a possible genetic involvement in the aetiology of the ROHHAD syndrome, although some studies ruled out the presence of mutations in several candidate genes [82,83]. Epigenetic mechanisms have been hypothesised in some reports on the discordant presentation of ROHHAD syndrome in monozygotic twins [84].

In conclusion, CCHS is one of the rare monogenetic disorders of central respiratory control associated with mutations of the PHOX2B gene. The discovery of the association between CCHS and the PHOX2B gene helped clinicians in the diagnostic work-up of the disorder. The screening for the *PHOX2B* mutations has been proposed as an integral part of genetic counselling and prenatal screening as well as a potential target for gene therapy [85].

*Sleep-related hypoxemia disorder* is characterised by arterial oxygen saturation during sleep ≤90% in children for ≥5 min, as documented by polysomnography, out-of-centre sleep testing or nocturnal oximetry without hypoventilation. Obstructive sleep apnoea or CSA may be present, but these are not believed to be the principal cause of hypoxemia. The underlying pathologies are airway or pulmonary parenchymal disease, chest wall disorder, pulmonary hypertension or neuromuscular disorders. Consequences include pulmonary artery hypertension, *cor pulmonale* or neurocognitive dysfunction. The genetic patterns related to sleep-related hypoxemia reflect those of the underlying inherited conditions, such as muscular dystrophies or cystic fibrosis [21].

## 5. Central Disorders of Hypersomnolence

This group of disorders includes conditions whose primary complaint is excessive daytime sleepiness [21].

*Narcolepsy* is characterised by daily periods of irrepressible need to sleep or daytime lapses into sleep. Other associated features are hypnagogic or hypnopompic hallucinations, sleep paralysis and the disruption of nocturnal sleep. The ICSD-3 indicate a Narcolepsy type 1 (NT1) diagnosis if one or both of the following are present: (1) cataplexy and a mean sleep latency ≤8 min and two or more sleep-onset REM periods on a Multiple Sleep Latency Test; (2) the cerebrospinal fluid (CSF) hypocretin-1 concentration is either ≤110 pg/or <1/3 of the mean value obtained in normal individuals with the same standardised assay. Narcolepsy type 2 (NT2) is characterised by the absence of cataplexy with a CSF hypocretin-1 concentration >110 pg/or >1/3 of the mean value obtained in normal individuals with the same standardised assay [21]. It should be considered that, in children with severe depression, excessive daytime sleepiness can be reported. In these cases, CSF hypocretin can be decreased without sleep-onset REM periods on a Multiple Sleep Latency Test. Therefore, diagnosing narcolepsy involves an accurate clinical history (including a good psychiatric history) next to a number of tests performed under carefully controlled conditions. Cases with incongruous results benefit from review and re-testing after an interval of several months or longer [86].

Narcolepsy incidence is 0.83 per 100,000 person–years in children, pre-adolescents and adolescents aged 5 to 19 years [87]. Contrary to what happens with adults, in children the naps tend to be longer (30 to 90 min) and not consistently followed by a refreshed feeling [88]. Cataplexy may often present with facial hypotonia characterised by droopy eyelids, mouth opening, tongue protrusion or gait unsteadiness [89]. Depression, aggressive behaviours, attentional difficulties with school-related learning problems, impaired quality of life, obesity and precocious puberty are associated with the disorder [90,91,92,93]. The majority of cases of narcolepsy are sporadic, but cases of familial narcolepsy have been reported [94]. In some familial cases, mutations in genes modulating the immune response were found, such as the *genes encoding orexin* (*HCRT*), *myelin oligodendrocyte glycoprotein* (*MOG*) and *P2Y purinoceptor 11* (*P2RY11*) [95,96,97]. Family and twin studies indicate that narcolepsy arises from an interaction between the environment and a predisposed genetic substrate [98]. This substrate is represented by the human leukocyte antigen (HLA) class II region, which encodes molecules that present antigenic peptides to CD4+ T cells. HLA DQB1*0602 and DQA1*0102 are the primary susceptibility alleles for narcolepsy predisposition, although other HLA class II alleles can be encountered in patients with narcolepsy [99,100,101,102,103]. Considering that HLA-DQB1*06:02 is expressed in 86–98% of patients with NT1, in 40–50% of patients with NT2 [97], but also in 5–38% of the general population [101,102], the presence of a histocompatibility antigen is, per se, insufficient to precipitate narcolepsy. Human narcolepsy is therefore best explained by a two-threshold hypothesis, with an interplay between genetic susceptibility (in which the immune system is involved) and environmental factors such as major life events, systemic illness or injury [104]. GWAS provided further evidence of polymorphisms in genes involved in the immune response, such as the *gene-encoding T-cell receptor α-constant domain* (*TRAC*) [105] or the *gene-encoding tumour necrosis factor ligand superfamily member 4* (*TNFSF4*) [106].

The *Kleine–Levin Syndrome* (*KLS*) is characterised by recurrent episodes of excessive sleepiness and sleep duration persisting from two days to five weeks, in association with at least one of these symptoms: cognitive dysfunction, altered perception, eating disorder or disinhibited behaviour. The episodes usually recur more than once a year and at least every 18 months. The patient has a normal alertness, cognitive function, behaviour and mood between the episodes [21]. The prevalence of the disorder is estimated at around 1 to 2 cases per million. Adolescence (second decade) is the usual age of onset [107,108]. Diagnosis is mainly clinical [109]; it is necessary to exclude other disorders that can mimic its symptoms, such as structural insults of the central nervous system, encephalitis and toxic, metabolic or psychiatric disorders [21]. Social and occupational complications, long-term memory deficits and a higher risk of psychiatric disorders are encountered in patients affected [21]. Most cases of KLS are sporadic; familial occurrence (3 to 8% of relatives) has been reported in some studies [110,111], with some reports of concordant twin pairs suggesting genetic or shared environmental effects [111,112]. An autoimmune mechanism may confer susceptibility to the development of the disorder, as reported in a study on 30 patients with KLS in which an HLA-DQB1*0201 frequency was 28.3% [109]. A worldwide case-control GWAS in 673 KLS cases and ethnically matched 15,341 controls found a strong significant association within the 3′region of the *Tetratricopeptide Repeat And Ankyrin Repeat Containing 1* (*TRANK1*) gene locus, previously associated with bipolar disorder and schizophrenia. Pathway analysis also revealed the involvement of circadian regulation genes. This study also showed that variants in the *TRANK1* gene region may predispose to KLS in participants with a difficult birth, suggesting that the *TRANK1* gene region modulates newborns’ response to brain injury, resulting in mental and neurological health consequences [113].

In summary, Central Disorders of Hypersomnolence are HLA-associated, multigenic and environmentally influenced conditions. In narcolepsy, the utility of HLA genotyping as a diagnostic tool is controversial and the clinical history next to the Multiple Sleep Latency Test and the hypocretin measurement remain the gold standard for the diagnosis.

## 6. Circadian Rhythm Sleep-Wake Disorders

The circadian system is a complex, endogenous and essential organisation of biological rhythms, whose oscillations are entrained to the 24-h light-dark cycle. In the mammalian circadian system, this internal clock is genetically determined, self-perpetuating and located in the suprachiasmatic nucleus of the hypothalamus. The master clock must dynamically coordinate and adapt to both dark-light regulation and the body’s internal signals, in order to produce a functional network responding to environmental demand [19,21,114,115,116]. The disruption of the internal clock organisation or a mismatch between the individual’s circadian sleep-wake propensity and the 24-h environmental influences might induce a Circadian Rhythm Sleep-Wake Disorder (CRSWD). For the diagnosis of a CRSWD, the individual must also complain of insomnia, daytime sleepiness or both and be in significant distress due to an impairment in mental, physical and social areas of normal functioning [21].

**Figure 2:**
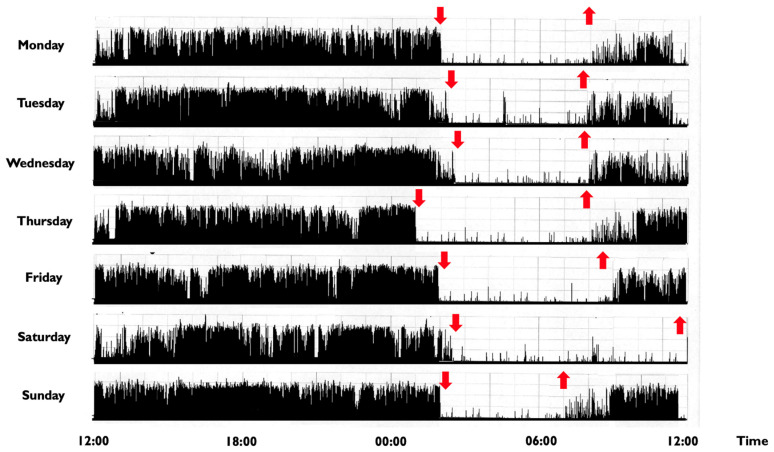
Representative rest activity cycles recorded in a 9-year-old boy with a delayed sleep phase disorder. **Note**: The actigraphic recording shows a delayed sleep-wake phase disorder with sleep onset times at around 1:30–2:00 a.m. and wake times at 8:00 during the week and at noon during weekends (the black bars indicate wake activity levels recorded at the non-dominant wrist). Downward red arrows indicate the sleep onset, while upward red arrows indicate the awakening. The actigraph model used is Micro MotionloggerWatch (Ambulatory Monitoring, Inc., Ardsley, NY, USA).

In children and adolescents, a CRSWD might occur in about 10–18% of individuals [2], with a prevalence for the *Delayed Sleep-Wake Phase Disorder* (DSPD) [117]. The DSPD involves difficulty falling asleep at the desired bedtime, producing a significant delay in the major sleep episode, as well as difficulty awakening at the required clock time (Figure 2) [21].

In addition to clinical history, the diagnosis may benefit from a sleep diary and actigraphic recordings, while a VPSG is not required, unless a comorbid sleep disorder is suspected. In DSPD, a positive family history is reported in about 40% of individuals and polymorphisms in some genes, such as *PER3*, *CLOCK* and *arylalkylamine n-acetyltransferase* have been suggested to play a role in the disorder [18,21,118]. The *PER3* gene is of particular interest because it causes opposite phenotypes depending on the length of a common variable number tandem repeat (VNTR) polymorphism in the coding region [13]. Human carriers of the long allele (five repetitions of the VNTR) were found to be associated with an extreme morning preference, while the shorter allele was related to extreme evening preference and DSPD [118,119,120]. Moreover, carriers of the long allele displayed increased slow-wave sleep, theta and frontal delta activity, as well as a more pronounced detrimental effect of sleep deprivation, indicating the important role of this variant also in sleep structure and homeostasis [5,13,18,32]. *CLOCK* mutations/polymorphisms might be associated with the evening preference, as suggested by animal models in which a *CLOCK* gene mutation led to phase delays of the rhythm for body temperature, locomotor activity and wake duration [120]. In 2017, a case of familial DSPD linked to a mutation in *cryptochrome circadian clock 1* (*CRY1*) has been reported [121]. *CRY1* is one of the main transcriptional inhibitors in the negative feedback loop of the molecular circadian clock, representing a critical regulator of circadian period length.

Contrary to DSPD, the *Advanced Sleep Phase Disorder* (ASPD) is characterised by the earlier occurrence of the major sleep period due to the difficulty in staying awake until the desired bedtime along with early morning awakening [21]. The ASPD is less frequent than DSPD in paediatric populations [2]; a correlation with preterm birth has been demonstrated [122,123]. Children or adolescents with ASPD fall asleep immediately after or even before dinner, during public events or schoolwork [2]. Familial cases have been described, and the *Familial Advanced Sleep Phase Disorder* (FASPD) is recognised as a heritable phenotype of the disorder, with few genetic mutations described so far [18,121,124,125]. In the first FASPD family, a mutation of the *PER2* gene was identified [124]. This mutation produced an alteration in the *PER2* phosphorylation state, responsible for an acceleration of the circadian period [119,120]. Similarly, the second causative mutation for FASPD was encountered in *human Casein Kinase I delta* (*CKIδ*), an essential core component of the circadian clock involved in the phosphorylation of PER proteins. By altering its function of phosphorylating PER proteins, the mutation caused an acceleration of the 24h period and advanced sleep phases [119,120,125]. In another family, a missense mutation in the *human Cryptochrome 2* (*CRY2*) gene has been described. This mutation alters the conformation of *CRY2*, leading to an easier degradation by a ubiquitin complex, causing a shortened circadian period with phase-advanced behavioural rhythms [126]. Interestingly, two rare variants in the gene *PER3* were identified in a family with FASPD, whose individuals also displayed high depressive and seasonality scores. The authors suggested that *PER3* might constitute a link between sleep and mood disorders, especially in the refinement of these two processes to adapt to periodic seasonal changes [127]. Finally, a study researching genes for the “extreme early bird” phenotype disclosed a mutation in the *human DEC2 gene* (*hDEC2*) [128]. *DEC2* is a transcription factor regulating the circadian clock in mammals, although its definite role in sleep regulation has not been completely elucidated [129]. The described individuals carrying the mutation exhibited a “Natural Short Sleeper” phenotype with a lifelong daily sleep time need of about 6 h [120,128]. Another variant of this gene mutation showed, in humans, a short sleep phenotype together with resistance to sleep deprivation, suggesting that, beyond the circadian clock system, this gene might be implied in sleep homeostatic processes [130].

In addition, given the complexity of the signalling pathways involved in the circadian clock gene system, an increasingly expanding ground for genetic studies has been flourishing over recent years, with special attention directed at chronotype [18,119,120,131]. So far, slightly more than 300 chronotype-associated loci have been identified from GWAS [18,132,133]. Together with more predictable variants of genes of the circadian clock core, other variants affecting different physiologic pathways were linked to the individual chronotype and included: variants related to the development and functioning of retinal ganglion cells; variants in genes regulating appetite or insulin secretion; variants involved in non-essential habits such as nicotine and caffeine metabolism [133].

In summary, an alteration of circadian system genes appears to be associated with a wide variety of conditions, going from multiple sleep disorders (insomnia, sleep breathing disorders etc.) to the alteration of sleep homeostasis and various medical conditions, such as cardio-metabolic disturbances or mood disorders. As a consequence, a body of evidence is growing up relating the evening chronotype or a DSPD to neurodevelopmental disorders and mood disturbances, including major depression, bipolar disorder and schizophrenia [20,133]. Complex sleep phenotypes, involving different types of circadian disturbances, have been described in attention-deficit hyperactivity disorder, autism spectrum disorders and some genetic conditions, like the Prader–Willi and Smith–Magenis syndromes [20]. Improving the circadian system’s regulation might be helpful to improve the quality of life of these patients.

## 7. Parasomnias

Sleep parasomnias are currently defined as undesirable physical experiences occurring during the entry into sleep, within sleep or during arousal from sleep [21]. They may occur in NREM, REM sleep or during the transition from wakefulness to sleep or vice-versa [21].

**Figure 3:**
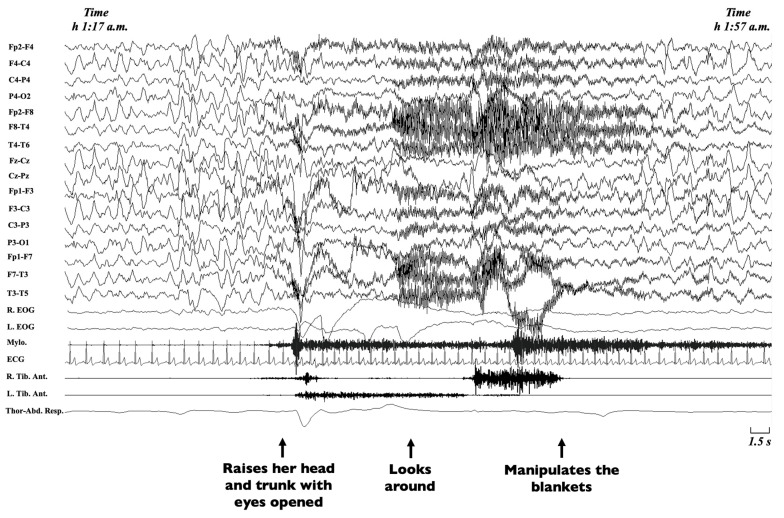
Excerpt of a polysomnographic tracing showing a confusional arousal in a 7-year-old girl. **Note**: The polysomnographic tracing shows an episode arising from stage-3 NREM sleep during which the patient raises her head and trunk with eyes opened, looks around and manipulates the blankets. The EEG leads before the episode shows diffuse delta waves typical of deep sleep, which persist during the episode, intermingled with movement artifacts. EEG, electroencephalogram (Fp2-F4; F4-C4; C4-P4; P4-O2; Fp2-F8; F8-T4; T4-T6; Fz-Cz; Cz-Pz; Fp1-F3; F3-C3; C3-P3; P3-O1; Fp1-F7; F7-T3; T3-T5); R, right; L, left; EOG, electrooculogram; Mylo., mylohyoideus; Tib. Ant., tibialis anterior muscle; ECG, electrocardiogram; Thor-Abd. Resp., thoraco-abdominal respirogram.

Non-rapid eye movement (NREM) sleep parasomnias are a group of motor manifestations characterised by the occurrence of incomplete awakenings from NREM sleep. This group currently includes the *Disorders of Arousal* (DoA) and the *Sleep-Related Eating Disorder* (SRED) [21]. Traditionally, familial recurrence is recognised in SRED, but no systematic genetic studies have been performed [21], while DoA have been more widely studied. DoA are frequent in childhood and adolescence, encompassing three main clinical entities, namely confusional arousals, sleep terrors and sleepwalking [134,135]. Confusional arousals are characterised by a brief awakening from sleep, sitting up in bed, looking around and returning to sleep (Figure 3) [2].

During terrors, children rise abruptly from bed, scream or cry inconsolably, with eyes open and an expression of intense fear [2,136]. In sleepwalking, children may awaken in a different room, wander to their parents’ room or behave inappropriately [2]. To date, a diagnosis is performed on a clinical basis following the ICSD-3 criteria, including the incomplete arousal from sleep, an absent or incongruous response to the external attempts of awakening, a variable or no dream content, amnesia regarding the event and the exclusion of other causes [21]. In atypical cases or to rule out a suspicion of seizures, a VPSG should be performed [137]. Since the earliest descriptions, a strong genetic component has been recognised in DoA [138]. Different studies have shown a higher likelihood of DoA in monozygotic rather than dizygotic twins as well as a higher probability of sleepwalking if at least a parent had presented a DoA in infancy [139,140,141,142,143,144,145,146]. Still, no genes have been identified in family pedigrees, and the most probable mode of inheritance is considered to be multifactorial [141]. The closest identification of a single gene comes from a four-generation family composed of 22 individuals, of which nine were affected by sleepwalking, and in which a linkage to chromosome 20q12-q13.12 was identified. Among the 28 genes from the exonic sequence involved, the *Adenosine Deaminase gene* (*ADA*), associated with the quantity of slow wave sleep (SWS), was considered the most likely candidate [147], but no mutation was disclosed. Other studies analysing the relative contribution of genetic and shared and non-shared environmental factors disclosed a double contribution given by genetic factors, on the one hand, accounting for 44%, and non-shared environmental factors for the remaining 56%, on the other [148]. In addition, a higher genetic susceptibility for DoA has been demonstrated in individuals carrying the DQB1*0501 HLA haplotype, a type that is different from the one encountered in narcolepsy [149,150], suggesting a possible involvement of immune-related mechanisms in motor control during sleep. Finally, a specific demonstrated heritability also for homeostatic factors of sleep, together with an EEG trait predisposition, might further account for genetic factors in DoA [9,151].

*REM sleep parasomnias* include *REM Sleep Behaviour Disorder* (RBD), *Recurrent Isolated Sleep Paralyses* and *Nightmares*. RBD is characterised by vocalisation or complex motor behaviour, mimicking a dream enactment and requiring VPSG evidence of episodes arising from REM sleep or REM sleep without atonia [21]. This parasomnia is currently considered a prodromal phase of alpha-synucleinopathy and occurs typically in adulthood [152]. In paediatric populations, the reports of RBD are mostly anecdotical and related to structural lesions, neurodevelopmental disorders or rare conditions, like the Smith–Magenis and Moebius syndromes [2,153,154,155,156]. More consistently, RBD occurs in paediatric narcolepsy, where it can even be the presenting symptom [157,158].

*Sleep paralysis* is characterised by the inability to perform voluntary movements at the transition to (hypnagogic) and from (hypnopompic) sleep, lasting seconds or minutes and typically accompanied by an anguishing sensation [21]. When recurrent, they might provoke significant distress and anxiety during sleep time. Other conditions, especially a diagnosis of narcolepsy, have to be excluded [21]. Familial occurrence has been described in some families [159], but, to date, a single twin study disclosed an association with the *PER2* circadian gene, which, however, did not survive the Bonferroni correction [160]. Another study failed to identify an association of sleep paralysis with HLA commonly involved in narcolepsy [161].

*Nightmares* are unpleasant, dysphoric dreams, which lead the individual to awaken in full alertness and report a vivid and detailed recall of the content, without any “dream-enacting” motor activity [162]. To be diagnosed as a disorder, nightmares must be perpetuated and induce a significant impairment in social, occupational or other life aspects of the individual [21]. Nightmares are frequent in children, being an occasional finding in up to 75% of them [21], but persisting as a disorder only in 5% of them, according to a recent meta-analysis [163]. Some twin studies showed a high heritability of nightmares [164,165], but no genetic loci have been identified up to now.

Among other parasomnias, *sleep enuresis* is characterised by recurrent involuntary voiding during sleep (at least twice a week) for at least 3 months, in children older than 5 years. It further subdivides into primary and secondary, depending on whether the child has never been dry or has been dry for at least 6 months [21]. It is the most frequent urinary symptom in the paediatric field, affecting around 6 million children in the United States [2]. The pathogenesis is complex and probably related to the interplay of multiple factors, such as a high nocturnal production of urine, detrusor overactivity and a reduced arousability from sleep [2,21]. Since the first descriptions, a high heritability has been highlighted in sleep enuresis [166]. Both an autosomal dominant and a recessive mode of inheritance have been described [167]. Some loci were discovered in chromosomes 6, 8, 12, 13 and 22 [167,168,169], involving genes associated with sleep process, urine production and bladder function [169].

Sleep parasomnias, with the exception of adult RBD, are mostly unusual physiological phenomena, which normally do not need pharmacological treatment. Consequently, genetic studies are lacking for this group of disorders, although a clear genetic background is well recognised for some of them. Sleep parasomnias emerge from defective mechanisms controlling the transitions between different sleep and wake stages. Enhancing the knowledge in this particular field could thus help to understand the neurophysiological mechanisms regulating sleep.

## 8. Sleep-Related Movement Disorders

Sleep-related movement disorders are characterised by simple, stereotyped movements usually affecting sleep or its onset, with the exception of the *Restless Leg Syndrome* (RLS), which involves deambulation or non-stereotypical movements to reduce leg discomfort [21].

*RLS* is characterised by: (1) an overwhelming urge to move legs (or other body parts) usually associated with an unpleasant sensation, (2) which begins or worsens at rest, (3) which could be temporarily relieved with movement (4) and occurs predominantly in the evening or night [170]. In children, in addition, the diagnosis of RLS requires the description of discomfort in the child’s own words or two of the following features: (i) a positive family history; (ii) the documentation of periodic limb movements of the sleep (PLMS) index >5/h at VPSG; (iii) sleep disturbance for age [171]. The prevalence rate of definite RLS in childhood is nearly 2%; up to 40% of adults affected by RLS report the onset of symptoms in childhood or adolescence [172,173]. In children, RLS may be idiopathic, with a strong familial recurrence (heritability of around 70% estimated by twin studies [174,175]) with a variable phenotypic expression and the possibility of anticipation [176,177,178]. RLS may be present in conditions such as iron deficiency, Sickle cell disease, Celiac disease, Osgood–Schlatter disease, Diabetes and Thyroid disease [2]. GWAS in RLS identified several risk loci with functions spanning from neurogenesis (*MDGA1*, *MYT1*, *NTNG1*, *SEMA6D*), cell-junction organisation (*PKP4* and *SMAD3*) and axon guidance (*NTNG1* and *SEMA6D*) to DNA repair/maintenance (*APLF*, *ASTE1*, *DIS3*, *PRMT6*, and *RNF8*) and locomotor behaviour (*BTBD9*, *CLN6*, *HOXB8*, and *MEIS1*) [42,179,180]. Winkelmann et al. were the first to identify the strongest genetic risk factor for RLS in the single nucleotide polymorphisms (SNPs) in the intronics, and rarely coding variants of *MEIS1* locus, which encodes for a transcription factor involved in haematopoiesis but also in the neurodevelopment of the proximodistal limb axis [181]. A study specifically targeting childhood-onset RLS confirmed an increased frequency in their index cases of the risk allele in the *MEIS1* and the *LBXCOR1*/*MAP2K5* regions, while an association with the third susceptibility region (*BTBD9*) was excluded [182].

PLMS consist of involuntary recurrent and stereotyped limb movements arising from all sleep stages [65], usually characterised by a dorsiflexion of the foot and lower leg [183]. A higher PLM index could be an independent cause of sleep disturbance with pathological repercussions, representing the Periodic Limb Movement Disorder (PLMD) [21]. Children with PLMD have been reported to present unspecific symptoms such as pain, restless sleep and hyperactivity [184]. The diagnosis of PLMD in children and adolescents requires all the following features: (1) polysomnographic documentation of the PLM index >5 per hour, (2) clinical sleep disturbance, and (3) the absence of another primary sleep disorder (including RLS) [21]. The prevalence of PLMD in paediatric populations has been estimated at around 0.3% [185]. Genetic studies in adults identified some risk loci in PLMD independently of RLS [180,186], but specific data in the paediatric population are lacking.

*Sleep bruxism* (SB) is a repetitive jaw-muscle activity characterised by involuntary tooth grinding and jaw clenching during sleep. Children or adolescents may complain of transient morning tension headache, jaw muscle pain or jaw lock. Chronic sleep bruxism may provoke tooth wear damage and sleep fragmentation [21,187]. The prevalence in childhood is up to 40% [135]. A tooth-grinding sound during sleep reported by parents, associated with the presence of clinical symptoms/signs of alterations of the stomatognathic system (abnormal tooth wear, hypertrophy of the masseter muscles, discomfort/fatigue or pain in the jaw muscles) allow for a bruxism diagnosis. A video-polysomnography including an EMG recording of masticatory muscles is not routinely required in isolated SB [21]. Twin and familial aggregation studies showed a certain tendency of SB to occur in families [188,189]. The probable genetic component in SB genesis involves personality traits (with a high level of stress and anxiety) [190] and the dopaminergic/serotoninergic systems, as these neurotransmitters regulate the homeostasis of breathing, muscle tone, sleep, stress and reward [191]. Polymorphisms in the *gene coding for serotonin receptor 2A* (*HTR2A*) have been associated with SB [192], while SNPs in the dopaminergic pathways could be protective (Dopamine receptors D 2 and 5) or risk factors (Dopamine receptors D3), as they respectively increase or reduce the dopaminergic activity in the central nervous system [193].

*Sleep-related rhythmic movements* (SRRMs) are repetitive, stereotyped and rhythmic motor behaviours that occur while one is asleep or drowsy, involving large muscle groups. The sleep-related rhythmic movements disorder (SRRMD) may entail significant clinical repercussions (such as sleep disruption, daytime function impairment or body injuries) [21]. SRRMs are characteristic of infants, reaching a prevalence of up to 60%, and usually resolve spontaneously during childhood [194]. A video-polysomnographic recording is required in atypical cases, to exclude possible mimics [194]. Although familial occurrence seems to be rare [195], a genetic predisposition to SRRMs/SRRMD has been proposed in some case reports [196,197,198,199].

Sleep-related movement disorders represent a wide group of manifestations. Many of them show a consistent familial aggregation, even if no specific inheritance patterns and genes have been identified, suggesting complex environmental and genetic interactions (i.e., both iron deficiency and specific genes’ polymorphisms were found to be associated with RLS).

## 9. Limitations and Conclusions

The genetic predisposition recognised for several sleep disorders has led to a growing body of scientific evidence depicting a complex influence of genetic factors in sleep mechanisms. In addition, highly heritable EEG and sleep traits involved in sleep homeostatic processes have been described and add further complexity to this picture [9,32,200].

Genetic factors play a role of variable weight among the different sleep disorders and show an interplay with the environment that must often be taken into account. From the most recent genetic studies, an intricate picture linking sleep disorders and neuropsychiatric conditions, on the one hand, and metabolic profile, on the other, is emerging [18]. Therefore, expanding the current knowledge on this topic is essential to deepen our understanding of the fascinating variety of sleep functions and their influence on physiological processes, in order to develop possible targeted therapies for these different conditions.

As the main limitation of our paper, we have not performed a quality assessment of the included references, as usually performed for systematic reviews. However, we made a considerable effort to illustrate and synthetise the principal genetic aspects in all paediatric sleep disorders, trying to cover the whole field.

## Data Availability

Not applicable.

## Glossary

International Classification of Sleep Disorders—Third Edition (ICSD-3); Genome-Wide Association Studies (GWAS); Restless Leg Syndrome (RLS); Obstructive Sleep Apnea Syndrome (OSAS); Central sleep apnea (CSA); Congenital Central Alveolar Hypoventilation Syndrome (CCHS); Late-Onset Central Hypoventilation with Hypothalamic Dysfunction (ROHHAD); Narcolepsy Type 1 (NT1); Cerebrospinal Fluid (CSF); Narcolepsy Type 2 (NT2); Human Leukocyte Antigen (HLA); Kleine–Levin Syndrome (KLS); Circadian Rhythm Sleep-Wake Disorder (CRSWD); Delayed Sleep-Wake Phase Disorder (DSPD); Variable Number Tandem Repeat (VNTR); Advanced Sleep Phase Disorder (ASPD); Familial Advanced Sleep Phase Disorder (FASPD); Disorders of Arousal (DoA); Sleep-Related Eating Disorder (SRED); REM Sleep Behaviour Disorder (RBD); Single Nucleotide Polymorphisms (SNPs); Periodic Limb Movements of Sleep (PLMS); Periodic Limb Movement Disorder (PLMD); Sleep Bruxism (SB); Sleep-Related Rhythmic Movements (SRRMs); Sleep-Related Rhythmic Movements Disorder (SRRMD).
